# AI sees an end to filariasis

**DOI:** 10.1371/journal.pntd.0012260

**Published:** 2024-07-11

**Authors:** Michael W. Gaunt, J. Lee Crainey

**Affiliations:** 1 Solena Ag., Foster City, California, United States of America; 2 Instituto Leônidas e Maria Deane, Fundação Oswaldo Cruz (Fiocruz), Adrianópolis, Manaus, Amazonas, Brasil; National Institutes of Health, UNITED STATES

The WHO has successfully controlled lymphatic filariasis and onchocerciasis transmission with mass drug administration (MDA) in almost all the areas it has targeted [1,2]. The success of Latin American filarial disease control programs means the region will soon be free of both onchocerciasis and lymphatic filariasis; however, the elimination of these diseases in Africa and the control and elimination of other filarial diseases, like loiasis and mansonellosis, will require blood surveys in regions with little or no base-line surveillance data [3–7]. Historical expansions of filarial disease interventions required significant investment in specialist training for the collection of reliable surveillance data and thus the new digital technology reported by Lin and colleagues in this journal is a potential game changer [7–9].

Imagine a future where training for 100% reliable filariasis diagnosis is as simple as downloading an app and connecting a cellphone to a microscope eyepiece. Lin and colleagues describe a computer vision platform which to our knowledge is unprecedented for its filariasis surveillance potential: it’s an app available on Google Play, does not need specialist equipment or sample preparation, and there are no obvious barriers to its rapid roll-out [9].

In simple terms, the authors “trained” a neural network (NN) with over 2,000 microscopic images of thick blood smears from patients infected with *Mansonella perstans*, *Loa Loa*, *Wuchecria bancrofti*, and *Brugia malayi*. The authors informed the algorithm what species was in each image, and then let its “deep learning” work out the differences.

*Wucheceria bancrofti* and *B*. *malayi* are both current epidemiologic targets of WHO MDA interventions; however, the app’s ability to discriminate *M*. *perstans* and *L*. *loa* microfilariae is arguably of more interest to the WHO [6,7,9–11]. Almost all the WHO’s MDA in Africa is done with ivermectin doses that can be fatal for patients with heavy loiasis parasitic loads and thus close monitoring of loiasis infections is required in all ivermectin-based MDA operations [10–12]. There are currently around 186 million Africans infected with *M*. *perstans* and it is the most likely microfilariae to appear on blood smears and be misdiagnosed as *L*. *loa* in WHO intervention zones [13,14]. Thus, the app has significant epidemiological potential.

The Lin and colleagues [9] app is advanced tech that is evident by a de facto model for computer vision, convolution neural networks (CNN), used by the tech giants such as Facebook. CNN, for example, looks for differences at 2D “boundaries” then stores this “learning” to memory. The authors described their AI as showing good overall performance with species specific variation. The diagnostic species “precision”, which approximates to true positives versus false positives, was 95% to 99% for *L*. *loa*, 60% to 97% for *M*. *perstans*, 95% to 100% for *W*. *bancrofti*, and 59% to 67% for *B*. *malayi*. Thus, a high false positive rate is occurring for *B*. *malayi* diagnosis which is corrected by further AI training. In addition, the authors demonstrated the AI had high “recall” (“sensitivity”), approximating to true positives versus false negatives, with 92% to 100% for each species, excepting a value of 76% for *W*. *bancrofti*. Thus, if the AI identifies *W*. *bancrofti*, then that is a statistically significant observation, but will miss around one quarter of infections from this species. However, again deeper AI training will resolve the matter.

Under the hood, when the algorithm “sees” a thick-blood smear slide it has not encountered, it checks its “learning” to classify the unknown image and hence gives species diagnosis. Unlike human vision, small visual differences between filariae species are not a barrier to progress with CNN because it can readily turn “molehills” to “mountains” through “weighting” once the pattern is identified. Identifying different *Mansonella* microfilariae, while difficult for optical analysis, is unlikely to present a challenge to a future version of the app described by Lin and colleagues [9].

There is no immediate reason why the WHO would need an app capable of discriminating between *M*. *perstans* and *M rodhainii* in Africa, but an ability to distinguish between *M*. *perstans* from *M*. *ozzardi* infections in Latin America would be valuable [5]. *Mansonella perstans* is refractory to ivermectin therapy, while *M*. *ozzardi* is susceptible; thus, future mansonellosis control needs reliable diagnosis between these 2 parasites for intervention, planning, and monitoring [5,14].

Lin and colleagues [9] is not without shortcomings: a 2,000+ image data set is small for CNN; at least 3 filarial species have not been trained in the CNN model, most importantly *M*. *ozzardi*; and, finally, a 3D printer is required for the cellphone-microscope adaptor which might hinder roll-out in several areas of the world. This tech is still the future, for example, the computer vision in facial biometrics is present in most cellphones and highly regarded, indicating the future direction of microscopic diagnosis. The authors have kindly provided a guide on how to use it on YouTube [15].
